# Effects of Arbovirus Multi-Host Life Cycles on Dinucleotide and Codon Usage Patterns

**DOI:** 10.3390/v11070643

**Published:** 2019-07-12

**Authors:** Nicole R. Sexton, Gregory D. Ebel

**Affiliations:** Department of Microbiology, Immunology and Pathology, College of Veterinary Medicine and Biomedical Sciences, Colorado State University, Fort Collins, CO 80523, USA

**Keywords:** dinucleotide, codon, robustness, mosquito, arthropod-borne viruses, arbovirus, purifying selection, diversification

## Abstract

Arthropod-borne viruses (arboviruses) of vertebrates including dengue, zika, chikungunya, Rift Valley fever, and blue tongue viruses cause extensive morbidity and mortality in humans, agricultural animals, and wildlife across the globe. As obligate intercellular pathogens, arboviruses must be well adapted to the cellular and molecular environment of both their arthropod (invertebrate) and vertebrate hosts, which are vastly different due to hundreds of millions of years of separate evolution. Here we discuss the comparative pressures on arbovirus RNA genomes as a result of a dual host life cycle, focusing on pressures that do not alter amino acids. We summarize what is currently known about arboviral genetic composition, such as dinucleotide and codon usage, and how cyclical infection of vertebrate and invertebrate hosts results in different genetic profiles compared with single-host viruses. To serve as a comparison, we compile what is known about arthropod tRNA, dinucleotide, and codon usages and compare this with vertebrates. Additionally, we discuss the potential roles of genetic robustness in arboviral evolution and how it may vary from other viruses. Overall, both arthropod and vertebrate hosts influence the resulting genetic composition of arboviruses, but a great deal remains to be investigated.

## 1. Introduction

Viruses utilize host machinery in lieu of encoding all requirements for propagation. Therefore, viral propagation necessitates close interactions with host cells and fine-tuned co-evolution. For many viruses, such as measles virus, this has resulted in a restricted host range that includes only a few closely related species [1]. On the other side of the spectrum are arthropod-borne viruses (arboviruses). Arboviruses are mainly RNA viruses that are transmitted by arthropods to various secondary host species—often vertebrates [2]. RNA arboviruses belonging to the families *Flaviviridae*, *Togaviridae*, *Bunyaviridae*, *Reoviridae*, *Rhabdoviridae*, and *Orthomyxoviridae* [3] cause extensive morbidity and mortality throughout the world in humans and agricultural animals [4,5,6]. Additionally, many arboviruses such as chikungunya (CHIKV), West Nile (WNV), dengue (DENV), zika (ZIKV), Japanese encephalitis (JEV), Mayaro (MAYV), and Rift Valley fever (RVFV) viruses have emerged in new host species or in new geographical locations demonstrating their ability to adapt to new environments and hosts [3,7,8]. Due to their requirement for alternating replication, arboviruses thus face unique evolutionary pressures during their lifecycles that could be expected to limit fitness potentials, yet they constitute persistent and emerging health threats suggesting the possibility that frequent host shifts may instead facilitate their emergence in new environments.

Invertebrates and vertebrates are separated by more than 570 million years of evolution [8], and thus are highly divergent organisms with vastly different immune systems. Alternating replication in two distantly related hosts likely imposes distinct evolutionary pressures compared with single host viruses, resulting in different patterns of mutation accumulation, dinucleotide, and codon usage. Examples of these include CpG dinucleotide avoidance in vertebrates but not invertebrates and codon and dinucleotide usage correlating with host usage [9,10,11]. Understanding the unique pressures faced by these multi-host viruses is important for identifying countermeasures for one of the most important groups of infectious diseases. Most studies of evolutionary pressures have focused on the interaction of viral proteins with host machineries [12,13]. Increasingly however, the importance of viral RNA, including the primary coding sequence, in all aspects of viral survival and propagation is appreciated [14,15,16].

Dinucleotide and codon usage bias are markers left on viral genomes that demonstrate how natural selection acts directly on RNA to impact fitness. Examination of nucleotide sequence data suggests that selection on codon bias appears to be weak in most organismal and viral systems. However, experimentally introduced perturbations in codon usage have been shown to decrease viral fitness when introduced, at times to the point of nonviability [17,18,19,20]. Codon and dinucleotide usage vary significantly between vertebrates and invertebrates [21,22]. In this review we highlight some of the pressures placed on arbovirus nucleotide usage as a result of alternating replication in highly divergent organisms. We summarize what is known about dinucleotide and codon usage in hematophagous arthropods. Although important for a discussion of the environmental context in which arboviruses evolve, vertebrate drivers of dinucleotide and codon biases have been reviewed elsewhere, so will not be covered in this review except in comparison to arthropods. For excellent reviews on broad drivers of codon preference please see [23,24,25]. Additionally, we compare arbovirus dinucleotide and codon usages with that of host species, then discuss the results of experiments that have introduced altered codon usage in arboviruses. Finally, we discuss evolutionary strategies arboviruses may have evolved that differ from single host viruses, and future areas of investigation into arbovirus RNA evolution.

## 2. Evolutionary Pressures on Arboviruses Resulting from Replication in Multiple Hosts

Host-cycling by viruses requires adaptation to the cellular environments of divergent host types in order to perpetuate. Both vertebrates and invertebrates have robust innate immune systems and some pathways are shared between the two (e.g., the Toll and JAK/STAT pathways) [26,27,28,29]. However, vertebrates heavily utilize interferon as a downstream effector stimulated through pattern recognition receptors (PRRs) by pathogen-associated molecular patterns (PAMPs), as well as B and T cell mediated adaptive immunity, both of which arthropods lack [28,30]. On the other hand, arthropods rely heavily on RNA interference (RNAi), in particular the exogenous small interfering RNA (exo-siRNA) response, for antiviral defense [31]. Within vertebrates, after reaching initial evolutionary equilibrium following colonization of a new host, purifying selection drives arbovirus evolution to retain genomes best suited to avoid interferon stimulation by PRRs. Occasional positive selection would also be expected in order to escape adaptive immunity [13]. During arthropod infection, arboviral genomes might also be expected to undergo purifying selection to avoid recognition by PRRs. However, sequence diversification also occurs due to siRNA targeting of the most common virus sequences [32]. In fact, this expected divergence in evolutionary pressures is observed for WNV where infection of birds results in strong purifying selection, and infection of arthropods increases viral population diversity [32,33,34,35,36]. Although specific pressures may be conditional [37]. Alternating replication therefore clearly imposes distinct selective environments as arboviruses move from one host to another.

Arboviruses must replicate within host environments and interact with host resources; this includes binding host proteins, using available tRNAs, and folding RNA into functional secondary structures, all of which may radically vary in different hosts. Mosquito-borne flaviviruses (MBFVs) have duplicated stem-loop structures in the 3′UTR, and for DENV individual structures were shown to be essential to replication in either mosquito or mammalian cells, therefore, both structures are required for cyclical transmission between the two [38]. RNA secondary structures are important mediators of multiple biological processes for both hosts and viruses [16], yet mRNA folding may be host dependent [39]. MBFV stem-loop duplications could allow the virus to bind entirely different proteins in alternate hosts or it could be a result of differential RNA folding, with one stem-loop structure optimized for each host. It is possible that other similar RNA structures are also essential for arboviral use of both invertebrate and vertebrate hosts. The impacts of RNA secondary and tertiary structures during host shifts remain poorly understood but could provide insight into why some viruses can infect both vertebrates and invertebrates while others are specific to one group.

As a result of the unique evolutionary pressures imposed by alternating replication in vertebrates and arthropods, trade-offs could be expected to occur to maintain arbovirus viability in both hosts, with adaptation to one host resulting in decreased fitness in the other. The comparative effects of experimentally replicating arboviruses in single vs. multi-host cycles have been studied for CHIKV [40], DENV [41], WNV [33,34,42,43], St. Louis encephalitis (SLEV) [42], Sindbis (SINV) [44], RVFV [45], vesicular stomatitis (VSV) [46,47], and eastern equine encephalitis (EEEV) [48] viruses. Much of this work is reviewed in [49] and [50]. Surprisingly, results indicated that host alternation does not automatically limit the adaptive potential of arboviruses, yet consensus sequences remain strikingly consistent in nature over time. Combined, these studies largely support that purifying selection is the dominant evolutionary pressure for arboviruses and suggest the mutant swarm may play an important role in their adaptive potential [49,50]. Either arboviruses are extreme generalists, thereby avoiding dual-host induced trade-offs or they have evolved mechanisms to limit the effects of trade-offs from dual-host use. Single host passaging does, however, result in different accumulations of mutations, suggesting that there are varied host-driven pressures that select for divergent optimal arboviral evolution, and these often include both nonsynonymous and synonymous mutations [41,43,44,47,48]. Overall, arboviruses cyclically replicate in highly diverse environments with different evolutionary pressures present; their genomes largely remain constant yet are positioned to evolve rapidly when novel conditions arise. Together, this suggests that known arboviruses are highly adapted to their dual host use and have evolved mechanisms to overcome the challenges associated with replication in diverse host environments.

## 3. Codon Usage Bias in Hematophagous Arthropods

Dinucleotide preference and codon usage in arthropods, particularly hematophagous arthropods, is only beginning to be investigated. Data available for other groups of organisms show that (a) codon usage bias exists, (b) it has strong influences on protein expression and stability (which can be tissue dependent), and (c) is driven by a complex mix of biology including avoidance of common promoter sequences, modifications to nucleic acids such as deamination leading to CpG suppression, and proportions of specific tRNAs [23,24,25,51,52,53]. Codon usage is frequently associated with G+C content, dinucleotide prevalence, host tRNA profiles, protein expression levels, and immune strategies [25]. In order to identify genetic markers and link them to potential biological causes, well-annotated host genomes are required. Only three representative genomes are available for tick species and only one of these, *Ixodes scapularis*, has an annotated genome. Therefore, little is known about tick dinucleotide preferences, codon usage, and tRNA profiles; although this may be changing soon [54]. Currently, 28 mosquito species have fully sequenced reference genomes, of these there are six annotated genomes available: *Anopheles gambiae*, *Anopheles darlingi*, *Anopheles sinensis*, *Aedes aegypti*, *Aedes albopictus*, and *Culex quinquefasciatus* (NCBI Assembly). Analysis of these genomes demonstrate that, similar to other organisms, conserved proteins of mosquitoes show strong signs of codon usage bias and proteins with strong codon usage bias have increased expression levels [55]. Expression levels correlate with codon usage bias in nearly every system studied to date including the additional arthropods: *Gryllus bimaculatus* (cricket), *Oncopeltuds fasciatus* (milkweed bug), and *Parhyale hawainsis* (an amphipod crustacean), suggesting this trait is ubiquitous across species [56]. Additionally, unique to the mosquito lineage, is the reduced use of the AGG codon. Reduced use of AGG is likely due to an evolutionary history of alternative translation of AGG to lysine instead of the standard arginine (or the serine encoded in invertebrate mitochondria) [57]. Overall, codon usage bias exists in mosquito genomes and has similar correlations with gene expression as has been observed in other species.

**Figure 1:**
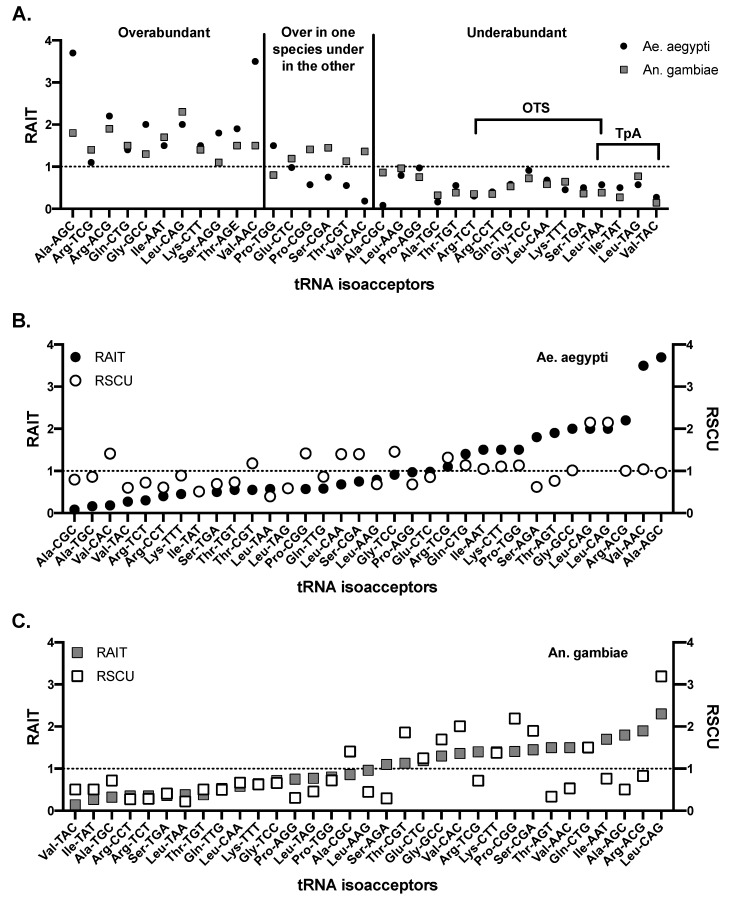
tRNA isoacceptor abundances in *Aedes aegypti* and *Anopheles gambiae* mosquitoes. (**A**) Relative abundance of isoacceptor tRNAs (RAIT) values for *Ae. aegypti* and *An. gambiae* mosquitoes. Underabundant tRNAs in both species that are likely due to avoidance of TpA dinucleotides or one-to-stop (OTS) codons are highlighted. RAIT values for (**B**) *Ae. aegypti* and (**C**) *An. gambiae* were plotted together with relative synonymous codon usage (RSCU) data. There is an imperfect correlation between the two relative abundances demonstrating the complexity of the tRNA-codon usage relationship. Data adapted from Behura and Severson (2011) [60].

Transfer RNAs (tRNAs) are host RNAs that assist in the translation of RNA to protein. tRNA include three nucleotide long anti-codon sequences that bind to RNA codons and facilitate the incorporation of the corresponding amino acid. Therefore, tRNA gene copies correlate with codon usage preferences in organisms, as would be expected given their role in translation [58,59]. Understanding the tRNA profiles of arthropods could be important to understanding pressures placed on arboviral RNA genomes via codon bias. *Ae. aegypti* and *An. gambiae* tRNA gene copies have been compared; *Ae. aegypti* encodes for 906 tRNA genes, nearly double the 441 found in *An. gambiae* [60] (613 are present in humans [61]). Overall, *Ae. aegypti* and *An. gambiae* have similar tRNA expression profiles, both in the percentage of tRNA devoted to specific amino acids as well as in similar under and overabundances of isoacceptors (tRNA utilizing different codons). Of the tRNA isoacceptors that are genetically underabundant in both *Ae. aegypti* and *An. gambiae,* many encode for codons that are TpA containing (see below) (e.g., valine-TAC isoacceptor resulting in the GTA codon with relative abundance of isoacceptor tRNAs (RAIT) scores of 0.27 in *Ae. aegypti* and 0.14 in *An. gambiae*, where 1 represents expected levels, >1 is overabundant and <1 is underabundant (Box 1)), or they are codons that are one mutation away from becoming a stop codon (a one-to-stop codon, OTS) (Figure 1) [18,60]. Both TpA containing codons and OTS codons are also avoided in vertebrates [9,21,62]. OTS codons are one mutation away from protein truncating non-sense mutations, therefore, are likely avoided to limit the generation of these lethal mutations. Mosquitoes do not avoid CpG dinucleotides or the codons containing them, which contrasts with strong CpG repression in vertebrates [9,60]. Many hypotheses exist to explain the avoidance of TpA dinucleotides including (a) avoiding TATA box and other protein binding sequences, (b) to optimize DNA stacking energy, (c) as a side-effect of avoiding UA-rich RNA regions resulting in mRNA decay, and (d) to avoid TAA and TAG stop codons [14,17,27,28,29]. Overabundant isoacceptors found in mosquitos can also be explained by TpA avoidance since, the overabundant isoacceptors counterbalance the underabundant isoacceptors. For example, *Ae. aegypti* and *An. gambiae* encode an overabundance of the Leu-CAG tRNA, allowing for few Leu-TAA or Leu-TAG tRNA genes, reflecting the avoidance of TpA containing codons. However, other abundances do not have clear correlatives, such as the underabundance of Ala-TGC, Arg-CCT, Gln-TTG, Lys-TTT and Thr-TGT, all with RAIT scores below 0.75 in both mosquito genomes (Figure 1) [60]. tRNAs that are underabundant in both *Ae. aegypti* and *An. gambiae* correlate with underabundant relative synonymous codon usage (RSCU) (Box 1) in all cases except for Leu-CAA (OTS) and Gly-TCC (OTS) in *Ae. aegypti*, and Ala-CGC in *An. gambiae* (Figure 1) [9]. However, correlation between preferred codons and highly abundant tRNA genes or the opposite is not universally observed for *Ae. aegypti* and *An. gambiae* [53,60,63]. Codon use in hosts, then, can be at least partially explained by differences in tRNA genetics and transcription.

Box 1 **Frequently used measures and scores for nucleotide and tRNA usages.**

**Box 1. Frequently used measures and scores for nucleotide and tRNA usages.** The usage of nucleotides across genomes is not random. Organisms show specific patterns of use for combinations of total nucleotides, pairs of nucleotides (dinucleotides), and for the specific codons used to select for amino acids. Additionally, these different nucleotide measures are intricately linked to each other. To better understand these biases many different measures have been developed. Those referenced in this review are summarized here:
**Relative abundance of isoacceptor tRNA (RAIT).** RAIT scores demonstrate preferences for specific isoacceptor tRNAs in a genome. RAIT scores are the ratio of observed isoacceptor tRNA gene copies over the expected copy number for a specific amino acid encoding tRNA group. Expected copy number is determined by the sum of all tRNA genes that result in identical amino acids, divided by the number of different tRNA isoacceptors resulting in that amino acid [60].
**Relative synonymous codon usage (RSCU).** RSCU was developed by Sharp and Li [64] and is used to determine how much bias is present in the use of codons for a particular amino acid. To calculate this, the observed number of a particular codon is divided by the expected number of codons if there was no bias. The expected value is calculated as the total number of codons that result in the amino acid of the codon being investigated divided by the codon degeneracy (number of codons resulting in that amino acid).
  $$RSCU_{ij}=x_{ij}/\frac{1}{n_{i}}\overset{n_{i}}{\underset{j=1}{\sum }}x_{ij}$$
  A value of 1 represents no bias. In this review we consider values above 1.38 and below 0.62 to represent over and underabundant codons. RSCU can be calculated using CodonW [65] or ANACONDA software [60,66].
**Dinucleotide odds ratios.** Similar to RSCU, the dinucleotide odds ratios provide numerical values to the amount of bias for or against a specific dinucleotide within a genome. It is measured as the observed number of a specific dinucleotide pair divided by the number of each individual nucleotide that make up that pair multiplied together.
  For viruses: Dinucleotide odds ratioxy=fxyfxfy
  $$\mathrm{For}\;\mathrm{hosts}:\;Symmetrical\;dinucleotide\;odds\;ratio_{xy}=\frac{2\left(f_{xy}+f_{zw}\right)}{\left(f_{x}+f_{y}\right)\left(f_{z}+f_{w}\right)}$$
  Here, *f*_k_ (where k = *x*, *y*, *z* or *w*) represent frequency of mononucleotides in a sequence; *z* and *w* represent complementary nucleotides to *x*/*y*. Similarly, *f_xy_* and *f_zw_* represent the frequency of dinucleotides in the same sequence. Again, 1 represents no bias; values above 1.25 or below 0.75 are considered to be significantly over or underrepresented [21].
**Effective number of codons (ENC).** The ENC was introduced by Wright [67] and is a measure of how equally synonymous codons are being used.
  ENC=2+9F2+1F3+5F4+3F6
  *F*_2_, *F*_3_, *F*_4_, and *F*_6_ represent the probability that randomly chosen codons for a specific amino acid are identical. ENC calculation results in a value between 20, indicating only one codon is used per amino acid, and 61 indicating that all synonymous codons are being utilized equally. CodonW software can be used to calculate ENC [65].

Codon usage and tRNA isoacceptor availability are clearly associated, however tRNA biology is more complicated than exclusively functioning as cognate aminoacylation machinery. Wobble base-pairing allows for binding of tRNA other than those containing an exact anticodon match. Molecular modifications on tRNA result in altered translational fidelity, altered processivity, or incorporation of an alternate amino acid (mis-aminoacylation). Additionally, some tRNA isoacceptors participate in cell physiology beyond translation, such as preventing cell death, and thereby can influence metastasis in cancer. In drosophilids, as well as other eukaryotes, the tRNAs encoding for tyrosine, histidine, asparagine, and aspartic acid by the GUN anticodon can be modified by the addition of queuosine. Queuosine modifications are dependent on the acquisition of the nutrient quinine from bacteria and levels of this modification vary across developmental stages and species in *Drosophila*. Queuosine modifications of tRNAs result in altered kinetic competition between related tRNAs; the result being changes in the specific tRNAs used to decode a codon, and the frequency with which a non-encoded amino acid is incorporated [68]. Finally, fragmented tRNAs are prevalent in cells and serve functions outside of aminoacylation once fragmented [59,68,69]. For example fragmented tRNA can interfere with translation during stress through tiRNAs, and fragmented tRNAs (tfRNAs) interact with factors unassociated with translation [69]. Due to these factors, specific tRNA abundances do not directly predict codon usage preferences in organisms. However, taking into account the influence of tRNA modifications can improve the correlation between tRNA abundance and observed codon preferences [58,68,70]. Overall, stress responses and nutrient acquisition affect the tRNA microenvironment so, when encountered by viruses during different stages of infection, and within different hosts and tissues may impact virus codon usage and evolution.

During viral infection, knowing the life stages and tissues most likely to be infected and the tRNA modifications present during infection will be important in understanding the codon usage preferences selected for in these organisms. Additionally, the codon usage biases of genes highly expressed during infection differs from those that are expressed during an uninfected state, with less codon usage bias present during infection [71]. Similarly, tRNA pools are altered by interferon, and influenza A virus codon usage better matches interferon induced tRNA pools [71]. Therefore, the environment that selects for viral codon usage is likely different from the observed baseline for a host organism. There is still a great deal to uncover in the overall biology of tRNA modifications, particularly regarding tRNA use in hematophagous arthropods. Since differences in tRNA modifications are observed even between species and life-stages of *Drosophila*, what role specific tRNA modifications have for mosquitoes and ticks, and whether they will differ across life-stages will need to be determined in order to identify contributing factors to the codon usage in these animals and their implications for arthropod-borne viruses. Overall, codon usage defines evolutionary potential by shaping the available mutational landscape and reflects environmental pressures an organism or virus face.

## 4. Dinucleotide Preferences in Arboviruses, Comparisons with Single Host Viruses

Similar to what is described above for vertebrate and invertebrate viral hosts, avoidance of CpG and UpA dinucleotides are the main drivers of dinucleotide preferences in RNA viruses. Host cells have many mechanisms to target non-self nucleic acids, zinc-finger antiviral protein (ZAP)-mediated depletion of CpG rich viral RNA in vertebrates, and degradation of AU-rich RNA across species being some of the better known [15,27]. In most virus families studied, both CpG and UpA dinucleotides are avoided by RNA viruses including *Arteriviridae*, *Astroviridae*, *Calciviridae*, *Flaviviridae*, *Picornaviridae*, *Arenaviridae*, *Bunyaviridae*, *Bunya*-*Arena-like*, *Filoviridae*, *Orthomyxoviridae*, *Paramyxoviridae*, *Rhabdoviridae*, *Mononega-like*, and *Birnaviridae* families [9,10,62]. Only *Coronaviridae*, *Arenaviridae*, *Orthomyxoviridae*, and *Rhabdoviridae* demonstrate an underrepresentation of any other dinucleotides (UpC, GpU, GpU, and GpC respectively). Similarly, only a few families do not underrepresent UpA (*Coronaviridae*, *Dicistroviridae*, *Hepeviridae*, *Togaviridae*, and *Reoviridae*) or CpG (*Hepeviridae*, *Nodaviridae*, *Togaviridae*, and *Reoviridae*) dinucleotides [10,62]. Arthropod-borne viruses, specifically, have genetic compositions that equally represent most dinucleotide combinations. The main exceptions are the underrepresentation of UpA and CpG, and the corresponding overrepresentations of CpA and UpG.

Broadly, dinucleotide frequencies are consistent across viral families, and more predictive of viral family than viral host species. However, within viral families that include vector-borne species, dinucleotide usage can be predictive of a vector-borne life-cycle [62]. Vector-borne virus dinucleotide odds ratios (Box 1) group most closely with those of vertebrate hosts, since vertebrates underrepresent both CpG and UpA. The only exception being arboviruses belonging to the *Orbivirus* genus (Reoviridae), which are more similar in dinucleotide use to their arthropod hosts [10]. Orbiviruses are, in general, understudied [72], so the reason for this is unknown, but if orbiviruses block CpG targeting by vertebrate hosts this might result in a closer match with mosquito dinucleotide use. Vector-borne viruses may also be distinguishable based on the type of arthropod used, as mosquito-borne and tick-borne viruses vary in G+C content and CpG underabundances [9,73]. However, these have been combined in most studies. Broadly, arboviral dinucleotide use seems to be driven by cyclical transmission between vertebrates and invertebrates, with vertebrate CpG repression having the most dominant effect on viral dinucleotide use.

Sequence composition (i.e., codon and dinucleotide use) has been investigated most thoroughly for *Flaviviridae* compared with other arbovirus-including families. The *Flaviviridae* family of viruses provides an excellent system for comparing the influence of host species on viral nucleotide composition as it includes many different life-cycle strategies and many of the viruses cause disease, so the family is well studied. *Flaviviridae* includes vertebrate-specific (*Hepacivirus*, *Pestivirus*), insect-specific flaviviruses (ISFVs), and vector-borne viruses (MBFVs and tick-borne flaviviruses (TBFVs)). No known vector flaviviruses will not be discussed here since as a group they have indistinguishable genetic compositions to MBFVs [9,73,74], and several no known vector viruses have been found to infect arthropods or arthropod cell cultures suggesting some (or all) of these are actually vectored viruses [74]. Instead, *Hepacivirus* and *Pestivirus* genera will be used to compare vertebrate-specific and arthropod-borne life cycles influence on dinucleotide and codon usage.

When grouped by host usage, patterns emerge demonstrating that there are dinucleotide usage differences within *Flaviviridae* that correlate with host species infected. Specifically, all flaviviruses that infect vertebrate hosts, including ZIKV and WNV, have an underabundance of CpG dinucleotides, and only Pestiviruses show no repression of UpA dinucleotides, whereas, ISFVs only demonstrate an underabundance of UpA dinucleotides [9,73,75,76,77]. An invertebrate-specific life-cycle is considered ancestral in flaviviruses, demonstrating the strong pressure against CpG dinucleotides driven by vertebrate hosts [31]. Pestiviruses avoid CpG dinucleotides more stringently than all other flavivirus categories. In contrast, *Hepacivirus* CpG suppression is less than in either pestiviruses or arthropod-borne flaviviruses [9]. This suggests that hepaciviruses have evolved alternative methods to avoid host targeting of these dinucleotides compared with host-switching flaviviruses [78,79], consistent with a long history as vertebrate-infecting viruses. Together this further suggests that dinucleotide usage is driven by host species usage, but that viruses evolve variable strategies to circumvent host selection against dinucleotide use, resulting in different outcomes across viruses that infect similar hosts.

Identifying patterns of host use for arboviruses and arthropod-specific viruses may require careful categorization of host type. This is particularly notable for viruses that utilize Hexapoda and other invertebrate hosts, which have faster evolutionary rates than vertebrates [3,37,38,39], resulting in significant differences between genera. For example, members of the genus *Phlebotomous* (sandflies) underrepresent CpGs, whereas mosquitoes do not [10]. When assessing virus-host genotypic similarities to arthropods, Hexapoda tend to be grouped together, which may obfuscate identification of host driven genetic patterns on viruses. This may explain why the Hexapoda species group in Di Giallonardo et al. [62] resulted in different dinucleotide distributions compared to other studies [9,10,21]. Therefore, arthropod grouping needs to be carefully considered for in silico host determinations, and for overall analysis of the genetic pressures presented by the arthropod host. Still, overall, dinucleotide usage in arboviruses is dominated by evolutionary pressure from the vertebrate host.

## 5. Arbovirus Codon Usage is Driven by Host Association, but Does Not Mimic Host Codon Usage

**Figure 2:**
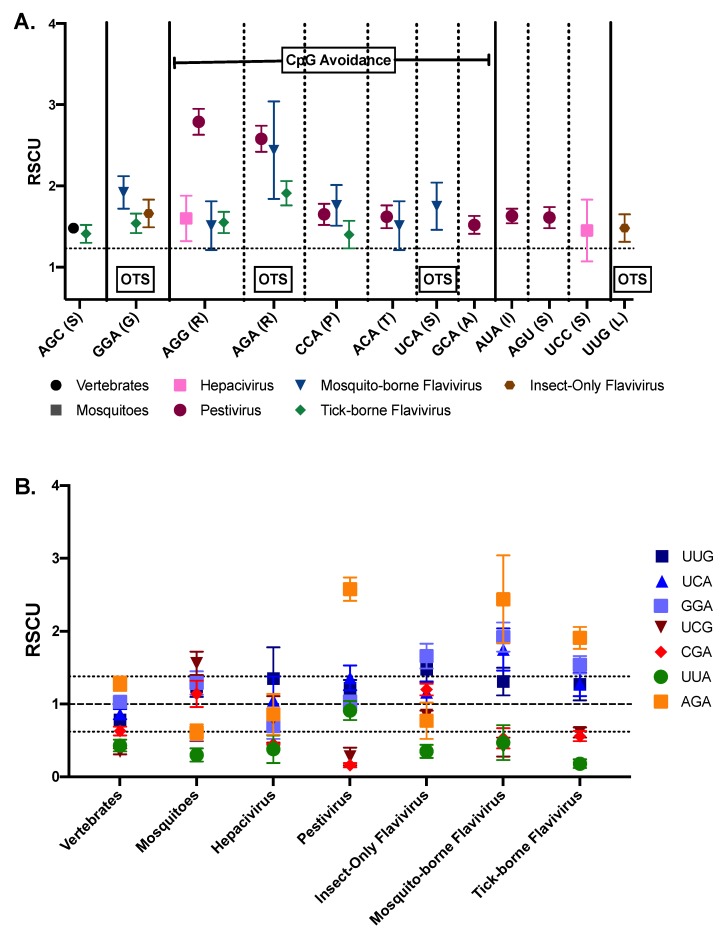
Over and underabundant codons in *Flaviviridae* and hosts. (**A**) Codons with RSCU values over 1.38 (dotted line) are shown for host and *Flaviviridae* viruses. Those that are likely the result of compensation for CpG avoidance are highlighted, as are OTS codons. (**B**) Gly, Arg, Ser, and Leu OTS codon usage is shown across *Flaviviridae* viruses and hosts. OTS codons are selected against in most systems investigated, likely to avoid lethal non-sense mutations, but are relatively abundant in insect-infecting flaviviruses. Dotted lines represent 1.38 and 0.62. Data adapted from Lobo et al. (2009) [9].

Given the dependence on host translational machineries, and specifically tRNA, one might expect viral codon usage bias to match that of the host. However, as described earlier in this review, tRNA expression differs during immune responses, based on nutrition, and between species [59,68,71,80]. Therefore, for many RNA viruses, and arboviruses in particular, codon bias does not correlate strongly with host codon use, this is true in Flaviviruses [9,76,77,81], Huaiyangshan (Bunyavirus) [82], and VSV (Rhabdovirus) [83]. For viruses infecting arthropods, the mosquito underabundant tRNA genes TGG, CCT, and TGT, all correspond with normal, or even elevated, representation of CCU, AGG, and ACA codons in insect infecting flaviviruses [9,60] (Figure 2A). However, hosts do influence synonymous codon usage of their viruses [10,73]. On average, arboviruses have high ENC values (52.0 on a 20–61 scale—Box 1) demonstrating that they use almost all available codons. However, for all arboviruses tested (except Bunyamwera virus (*Bunyaviridae*)) ENC values are significantly below that expected based on nucleotide composition alone, suggesting natural selection resulting in codon bias is occurring in vector-borne viruses [84].

As discussed in relation to dinucleotide usage, the *Flaviviridae* family provides a particularly compelling viral group to investigate the pressures on codon usage by host association due to the diversity of host usage within this viral family. Lobo et al. demonstrate that across flaviviruses the most striking differential patterns of codon usage are CpG and UpA dinucleotide driven [9]. However, this results in different codon usage patterns depending on genus and subgroups. All viruses in the *Flaviviridae* family that infect vertebrates, exclusively or in addition to an invertebrate host, repress the use of CpG containing codons, however the hepaciviruses utilize the arginine encoding CGC and CGG codons at values slightly above a neutral RSCU of 1, and only CGA has a RSCU value below 0.62. Instead hepaciviruses are the only *Flaviviridae* to mimic the codon usage of vertebrates. Similarly, all *Flaviviridae* underutilize UpA ending codons similar to both vertebrate and mosquito host genomes, with the exceptions of pestiviruses, which do not show any significant underabundance of any UpA containing codons, and significantly overrepresent the codon AUA (Ile) (RSCU above 1.38) [9] (Figure 2A). Of the *Flaviviridae*, pestiviruses repress CpG containing codons most strongly, despite moderate GC contents of ~46% and 47% for classical swine fever viruses (CSFV—*Pestivirus*) and bovine viral diarrhea virus (BVDV—*Pestivirus*), respectively [85,86]. It is possible that there are varied mechanisms used by pestiviruses and hepaciviruses to avoid vertebrate host immune responses and that these are the key drivers to these different patterns of codon usage observed, but more investigation into the comparative immunology of the two *Flaviviridae* genera is required [87,88].

CpG and UpA underrepresentation also strongly influences MBFV and TBFV codon usage, but repression falls between that of *Pestivirus* and *Hepacivirus* codon usage, repressing both moderately rather than strongly underrepresenting CpG or UpA. In MBFVs only UUA (Leu) and GUA (Val) UpA ending codons are restricted; and for TBFVs, CGC (Arg) codons are not underabundant [9]. Vertebrate host use also results in an overabundance of some codons but these again are likely driven by the avoidance of CpG dinucleotides. Specifically, the only CpG deficient arginine codons, AGG and AGA, are overabundant in the vertebrate-infecting *Flaviviridae* and correlate with the degree of CpG repression. However, whereas pestiviruses use AGG and AGA equally, MBFVs and TBFVs prefer AGA over AGG, and hepaciviruses only overutilize AGG (Figure 2) [9]. AGA is an OTS codon, and AGG is avoided in mosquito species, so these factors may play a role in the observed differential selection. Similarly, CpA ending codons are also overrepresented in the high CpG repression pestiviruses, MBFVs and TBFVs (Figure 2) [9]. In general, codon use for viruses that infect vertebrates is driven by avoidance of CpG dinucleotides and this seems to supersede other evolutionary pressures on codon usage. Arthropod-borne flaviviruses have not evolved ways to escape CpG or UpA suppression.

Generally, the use of arthropod hosts results in less dramatic codon usage bias than the use of a vertebrate host, and no codons that are overrepresented in mosquitoes and not vertebrates have associated overabundant viral codons. ISFVs use all CpG containing codons at expected frequencies within amino acid groups, while UpA ending codons are the most underrepresented codons. UpA avoidance in ISFVs mimics what is observed in the mosquito host, however mosquitos have an overrepresentation of many CpG containing codons making the use in ISFVs low in relation [9]. After removing all codons that are overabundant in both vertebrates and mosquitoes, only one codon is overrepresented for all flaviviruses that infect arthropods: GGA (Gly) (Figure 2) [9]. Glycine is encoded by GGA, GGU, GGC, or GGG codons, so overrepresentation of the GGA codon does not correlate with the avoidance of UpA dinucleotides, suggesting that it may be separately arthropod host driven. Like AGA, GGA is also a OTS codon and the additional OTS codon, UUG, is elevated in all arthropod infecting flaviviruses (Figure 2B) [9]. There are four amino acids which can be encoded for by OTS codons as well as non-OTS codons, these are leucine, serine, arginine, and glycine (Supplementary Figure S1). Interestingly, MBFVs and TBFVs have RSCU scores above 1 for all except the CpG and UpA containing OTS codons (Figure 2B) Presumably, organisms and viruses would avoid OTS codons when possible as they are more likely to result in nonsense mutations during replication. Supporting this, increasing the OTS codons in CVB3 and influenza A viruses were attenuating whereas decreasing the number had no effect on the viruses [18]. The impact of altered OTS usage on arbovirus replication and fitness is currently a topic of active research.

## 6. Altering Codon Position and Pair Bias Results in Attenuation of Arthropod-Borne Viruses

The interdependence of mononucleotide, dinucleotide, and codon usage has made it difficult to make conclusions regarding the involvement of natural selection across these viral features. Multi-component analyses often identify mutational pressure as the key driver of viral nucleotide usage at all levels, and from this it is sometimes stated that nucleotide and codon usage in viruses is not driven by natural selection, but instead is simply an accident of mutational preferences [89,90,91]. If this were the case, one would expect codon-altered viruses to be indistinguishable from wild-type (WT) viruses. Alternately, viruses could have reached genetic equilibrium and mutational preferences may be selected to maintain optimal genetic composition [17]. Advances in reverse genetics systems and gene synthesis have allowed for the generation of full, or partial, viral genomes engineered with altered-codons, which can be used to determine the manner in which alterations in synonymous codon usage may affect viral replication. RNA viruses including poliovirus [92,93,94], coxsackie B3, influenza A [18,71], classical swine fever [95], HIV [15,96], and others have be engineered with altered synonymous codons resulting in attenuation and reduced capacity for replication in almost all cases [97], demonstrating that natural selection acts on “synonymous” mutations in these viral genomes.

Arboviruses have also been engineered with altered codons, these include DENV [19,98], ZIKV [99], VSV [100], CHIKV [101], and tick-borne encephalitis (TBEV) [20] viruses. A summary of all codon altered arbovirus experiments can be found in Table 1. Codon pair bias (CPB) was investigated for DENV, ZIKV, and VSV. To investigate CPB the envelope (env), helicase (NS3), and RNA-dependent RNA polymerase (RdRp)/methyltransferase (Mtase) (NS5); the env and NS1; or the RdRp (L gene) respectively were recoded. For DENV and ZIKV recoding was designed to minimize the use of codon pairs that matched those used by humans but maintain a similar CPB to that preferred in mosquitoes, all while maintaining the global codon frequency of WT viruses [19,98,99]. For both DENV and ZIKV, shifting CPB away from the human preference resulted in attenuation in vertebrate cell culture and in vivo systems [19,99]. ZIKV resulted in a slight decrease in titers in mosquito cell culture [99], but codon pair recoding did not result in any changes to the replication of DENV in mosquito cell culture or in vivo [98]. It is important to note however, when codon pair usage is altered it routinely alters dinucleotide usage making it difficult to determine if CPB or a change in dinucleotide usage is the cause of attenuation [102,103]. Further, it may be that RNA secondary or tertiary structure, or RNA modification patterns, were disrupted in engineered viruses. Disentangling the different ways in which RNA sequences can impact virus replication remains an ongoing challenge.

As would be expected based on the known avoidance of CpG and UpA dinucleotides in vertebrates, codon altered viruses which increase the prevalence of CpG or UpA dinucleotides result in attenuation in vertebrate species [15,93,104]. For both DENV and ZIKV, CpG and UpA dinucleotides were increased in the codon pair altered viruses [19,98,99]. Therefore, results suggest that an increase in CpG and UpA dinucleotides through codon pair rearrangements minimally affect replication in mosquitoes but are highly attenuating in vertebrates. Future studies are required to determine if codon pairs, positions, or usage are important to the ability of ZIKV or DENV to replicate in their vertebrate or invertebrate hosts.

For VSV, recoding was designed to either minimize or maximize the use of codon pairs preferred in humans, again while maintaining the codon frequency of WT viruses. Viruses with engineered low preference codon pairs were not recoverable, potentially due to the high CpG content resulting from these codon rearrangements. VSV engineered with human preference codon pairs resulted in slightly lower CpG dinucleotide frequencies compared to WT and no change in the number of UpA dinucleotides; yet this virus still was attenuated, demonstrating that codon position, or pairs, are important beyond CpG dinucleotide frequencies [100].

Experiments with CHIKV and TBEV investigated the role of codon positions while specifically taking into account overarching GC, codon usage, UpA, and CpG contents. Viruses were designed by randomly assigning synonymous codons except those that are rare in primates (CGU, CGC, CGA, CGG, UCG, CCG, GCG, ACG), which were maintained in both codon and position. CHIKV was engineered with recoded Mtase/guanylyltransferase (nsp1), RdRp (nsp4), env, nsp1/nsp4, nsp4/env, or nsp1/nsp4/env. Only nsp1/nsp4/env contained an overall UpA content (full genome) that was increased above the natural range of 132 available CHIKV sequences. However, UpA dinucleotides were increased for all six mutant viruses and these would necessarily be concentrated as they are contained within one, two, or three proteins [101]. Concentrated areas of CpG dinucleotides are the target of ZAP, so it is possible that an increase in localized UpA dinucleotides could similarly be a target of the host immune system and/or AU-mediated RNA degradation [15]. TBEV RdRp/Mtase (NS5) was recoded using the same parameters as CHIKV. However, only G+C and codon usage contents were assessed for changes due to the recoding; both remained similar to WT [20]. Despite having controlled for genomic character outside of codon positions, both recoded CHIKV and TBEV were highly attenuated in vertebrate cell culture and in mice, resulting in an inability to compete with WT viruses and decreased disease severity [20,101]. These results suggest that synonymous codon choice is important to virus replication in a position dependent manner and that codon usage is not simply a result of mutational pressure. Further, when the nsp1/nsp4/env recoded CHIKV was passaged 50 times on Vero, C636, or alternating between these two cell types, the replicative capacity of these viruses increased over WT in most cases. The exception was C636-passaged viruses, that lost fitness in Vero cells [101], suggesting vertebrate-specific fitness defects existed. Increased replicative fitness resulting from passaging of codon altered viruses demonstrates that natural selection can act on synonymous codon sites.

Decreasing replication kinetics as a result of codon position and dinucleotide content were shown to be consistently concentration dependent across DENV, ZIKV, and CHIKV: the greater the percentage of the genome that was codon altered, the greater the inhibition to replication. DENV recoding was only performed for individual proteins whereas ZIKV and CHIKV were also assessed through the recoding of multiple proteins within a single genome [98,99,101]. In mosquito cells, recoding of ZIKV and CHIKV resulted in decreased replication kinetics proportional to the percentage of the genome that was codon altered, similar to what was observed in vertebrate cells. Interestingly, when generated in mosquito cells, both ZIKV and CHIKV RNA copies remained constant between WT and codon altered viruses despite decreased titers, contrasting with decreased levels for both RNA copies and titers in vertebrate cells [99,101]. Combined, these data suggest that synonymous codon usage is important to replication of arbovirus in both vertebrate and invertebrate systems, but that the pressures placed on replication due to codon reassignments differ between these two hosts. Considering the extensive differences between these two systems, including differences in immune responses, this is not surprising. However, it will be interesting to see where these data lead and how codon altered viruses can be used as tools to better understand the differential pressures arthropod-borne viruses face as a result of multi-host replication.

## 7. Genetic Robustness (or Lack Thereof?) in the Context of Alternating Hosts

An active area of virus research is in understanding what allows a virus to maintain a constant phenotype in settings where its genome faces change, referred to as genetic robustness [105]. In higher organisms, genetic robustness often takes the form of redundancy of genes and pathways [106,107]. Compared to cellular organisms, RNA viruses are quite brittle with the majority of mutations being deleterious and often lethal [108,109]. RNA viruses utilize genetic economy resulting in almost no self-encoded genetic redundancy (except RNA structural redundancy [38,110,111]), and reliance on host proteins for many functions [112]. Therefore, relative genetic robustness in RNA viruses is largely driven by codon usage and large populations sizes [113,114,115,116]. For excellent reviews of RNA virus robustness see [117] or [118]. Nearly all studies of genetic robustness in RNA viruses to date have focused on single-host viruses. In the setting of a single host virus, decreased genetic robustness results in attenuation of the virus, whereas increased robustness has little effect [18,113,119]. Interestingly however, genetic robustness can vary across a genome with some proteins encoded with more genetic robustness than others [120,121], suggesting the benefits of genetic robustness may be context dependent. Further supporting this idea Stern et al. [122] suggest through a combination of computational and experimental evolution data (using high and low multiplicity of infection (MOI) to represent robustness) that robust populations are positioned to rapidly fix beneficial mutations, whereas brittle (low robustness) populations efficiently purge deleterious mutations. Further, brittle populations may have an advantage in adapting to new environments, whereas robust populations have an advantage in stable environments [122].

Arthropod-borne viruses are continually replicating in new environments; they transmit between invertebrates and vertebrates, and also traverse through multiple tissue-specific bottlenecks in the invertebrate host. Therefore, if brittle populations benefit viruses when emerging in new environments arboviruses could be expected to prefer brittle genomes (relative to other RNA viruses). Although this area of inquiry requires more investigation two studies support this possibility. First, in the only direct test of the ability of an arthropod-borne virus to adapt as a function of robustness, VSV was shown to adapt more readily to new cell types from an evolved brittle population compared with an evolved robust population [123]. Second, Venezuelan equine encephalitis virus (VEEV) with a mutated polymerase that alters the types of mutations generated, resulted in decreased specific infectivity and attenuation in mice, yet reduced the number of stop codons introduced, contrary to expectations [124]. Although the VEEV study was not designed to directly investigate robustness, it nonetheless suggests that VEEV may have a propensity toward the generation of stop codons during natural infection since alteration of the types of mutations generated (three different mutation profiles) resulted in approximately half as many stop codons generated [124]. This is consistent with the overabundance of OTS codons seen for arthropod infecting flaviviruses (Figure 2) [9]. A great deal of work remains to be done to understand preferences for and against genetic robustness across virus life-cycle styles, host-types, and tissues tropisms. Investigating genetic robustness in arboviruses could provide a more complete picture of the role of robustness in viral evolution.

## 8. Conclusions and Future Directions

Arboviruses efficiently replicate in two widely divergent hosts, vertebrates and arthropods. Given our anthropocentric view, vertebrates are more comprehensively studied than arthropods. However, to understand how arboviral threats persist and emerge we need to know what evolutionary pressures drive fitness in these important viruses of humans, including in arthropods. In this review, we focus on evolutionary pressures that are independent of amino acid changes and show that both vertebrate and invertebrate hosts are important in shaping arbovirus genomes. Together, it is clear that a dominant evolutionary pressure on the genetic composition of vertebrate-infecting viruses is the avoidance of CpG dinucleotides, and the associated host responses. However, even closely related viruses seem to have evolved variable strategies to avoid the vertebrate CpG targeting. Additionally, through comparisons between closely related viruses that infect different host groups, combined with results from viruses with altered codons, we present evidence demonstrating that codon usage is also influenced by the arthropod host.

A comprehensive picture of arbovirus evolution is essential to understanding what predisposes an arbovirus to emerge in new hosts. Much remains to be investigated in order to fully understand the contexts in which arboviruses evolve. For example, arthropod tRNA profiles during infection are not fully characterized, and the impacts of altered nutritional status on viral replication in hematophagous arthropods are currently unclear. Minimal data is available regarding dinucleotide usage and codon usage in tick genomes and how this relates to the viruses that utilize these arthropods. Additionally, tick-specific viruses that might provide informative comparisons to tick-borne viruses are lacking. Altering codon usage effects arbovirus fitness, even when CpG content is controlled, but its impact is host-dependent. The mechanism(s) for this remain to be elucidated. Similarly, the effects of altering arbovirus genetic robustness and the overall role of robustness in arbovirus evolution and adaptation remains to be fully explored experimentally. Immune evasion routinely leaves behind genetic markers on viruses. Therefore, investigations into arbovirus genetic markers and comparisons with single host-viruses provides an opportunity to identify new host processes and potential therapeutic targets. Moreover, codon and dinucleotide usage has experimentally demonstrable impacts on arbovirus biology and evolution, but much remains to be learned in order to fully understand how these characteristics function in the full context of virus replication in different hosts.

## Figures and Tables

**Table 1:** Arbrovirus altered codon viruses.

Virus	Family	Codon Altered Proteins	CpGs Controlled?	Replication Kinetics		Competitive Fitness		Attenuation		Refs
				Invert	Vert	Invert	Vert	Invert	Vert	
**DENV**	*Flavivirus*	Env, Helicase (NS3), RdRP/Mtase (NS5)	No	NC	DECR	-	-	No	Yes	[19,98]
**ZIKV**	*Flavivirus*	Env, NS1	No	DECR	DECR	-	-	-	Yes	[99]
**VSV**	*Rhabdovirus*	RdRP (L gene)	Yes	-	NC	-	-	-	Yes	[100]
**CHIKV**	*Togavirus*	Env, RdRP (nsp1+4)	Yes	DECR	DECR	DECR	DECR	-	-	[101]
**TBEV**	*Flavivirus*	RdRP/Mtase (NS5)	-	-	NC	-	DECR	-	Yes	[20]

Invert = invertebrate, Vert = vertebrate, “-“ = not assessed, NC = no change, DECR = decreased.

## Supplementary Materials

The following are available online at https://www.mdpi.com/1999-4915/11/7/643/s1, Figure S1: codon usage table with one-to-stop (OTS) codons.

## References

[1] Laksono B.M., de Vries R.D., McQuaid S., Duprex W.P., de Swart R.L. (2016). Measles Virus Host Invasion and Pathogenesis. Viruses.

[2] Kuno G., Mackenzie J.S., Junglen S., Hubálek Z., Plyusnin A., Gubler D.J. (2017). Vertebrate Reservoirs of Arboviruses: Myth, Synonym of Amplifier, or Reality?. Viruses.

[3] Weaver S.C., Reisen W.K. (2010). Present and future arboviral threats. Antiviral Res..

[4] Paixão E.S., Teixeira M.G., Rodrigues L.C. (2018). Zika, chikungunya and dengue: The causes and threats of new and re-emerging arboviral diseases. BMJ Glob. Health.

[5] Maclachlan N.J., Zientara S., Wilson W.C., Richt J.A., Savini G. (2019). Bluetongue and epizootic hemorrhagic disease viruses: Recent developments with these globally re-emerging arboviral infections of ruminants. Curr. Opin. Virol..

[6] Linthicum K.J., Britch S.C., Anyamba A. (2016). Rift Valley Fever: An Emerging Mosquito-Borne Disease. Annu. Rev. Entomol..

[7] Lednicky J., De Rochars V.M.B., Elbadry M., Loeb J., Telisma T., Chavannes S., Anilis G., Cella E., Ciccozzi M., Okech B. (2016). Mayaro Virus in Child with Acute Febrile Illness, Haiti, 2015. Emerg. Infect. Dis..

[8] Peterson K.J., Lyons J.B., Nowak K.S., Takacs C.M., Wargo M.J., McPeek M.A. (2004). Estimating metazoan divergence times with a molecular clock. Proc. Natl. Acad. Sci. USA.

[9] Lobo F.P., Mota B.E.F., Pena S.D.J., Azevedo V., Macedo A.M., Tauch A., Machado C.R., Franco G.R. (2009). Virus-Host Coevolution: Common Patterns of Nucleotide Motif Usage in Flaviviridae and Their Hosts. PLoS ONE.

[10] Velazquez-Salinas L., Zarate S., Eschbaumer M., Pereira Lobo F., Gladue D.P., Arzt J., Novella I.S., Rodriguez L.L. (2016). Selective Factors Associated with the Evolution of Codon Usage in Natural Populations of Arboviruses. PLoS ONE.

[11] Mavian C., Rife B.D., Dollar J.J., Cella E., Ciccozzi M., Prosperi M.C.F., Lednicky J., Morris J.G., Capua I., Salemi M. (2017). Emergence of recombinant Mayaro virus strains from the Amazon basin. Sci. Rep..

[12] Enard D., Cai L., Gwennap C., Petrov D.A. (2016). Viruses are a dominant driver of protein adaptation in mammals. Elife.

[13] Daugherty M.D., Malik H.S. (2012). Rules of engagement: Molecular insights from host-virus arms races. Annu. Rev. Genet..

[14] Lawrie D.S., Messer P.W., Hershberg R., Petrov D.A. (2013). Strong Purifying Selection at Synonymous Sites in D. melanogaster. PLoS Genet..

[15] Takata M.A., Gonçalves-Carneiro D., Zang T.M., Soll S.J., York A., Blanco-Melo D., Bieniasz P.D. (2017). CG dinucleotide suppression enables antiviral defence targeting non-self RNA. Nature.

[16] Smyth R.P., Negroni M., Lever A.M., Mak J., Kenyon J.C. (2018). RNA Structure—A Neglected Puppet Master for the Evolution of Virus and Host Immunity. Front. Immunol..

[17] Hershberg R., Petrov D.A. (2008). Selection on Codon Bias. Annu. Rev. Genet..

[18] Moratorio G., Henningsson R., Barbezange C., Carrau L., Bordería A.V., Blanc H., Beaucourt S., Poirier E.Z., Vallet T., Boussier J. (2017). Attenuation of RNA viruses by redirecting their evolution in sequence space. Nat. Microbiol..

[19] Shen S.H., Stauft C.B., Gorbatsevych O., Song Y., Ward C.B., Yurovsky A., Mueller S., Futcher B., Wimmer E. (2015). Large-scale recoding of an arbovirus genome to rebalance its insect versus mammalian preference. Proc. Natl. Acad. Sci. USA.

[20] de Fabritus L., Nougairède A., Aubry F., Gould E.A., de Lamballerie X. (2015). Attenuation of Tick-Borne Encephalitis Virus Using Large-Scale Random Codon Re-encoding. Plos Pathog..

[21] Karlin S., Mrázek J. (1997). Compositional differences within and between eukaryotic genomes. Proc. Natl. Acad. Sci. USA.

[22] Karlin S., Ladunga I., Blaisdell B.E. (1994). Heterogeneity of genomes: Measures and values. Proc. Natl. Acad. Sci. USA.

[23] Plotkin J.B., Kudla G. (2011). Synonymous but not the same: The causes and consequences of codon bias. Nat. Rev. Genet..

[24] Hanson G., Coller J. (2017). Codon optimality, bias and usage in translation and mRNA decay. Nat. Rev. Mol. Cell Biol..

[25] Supek F. (2015). The Code of Silence: Widespread Associations Between Synonymous Codon Biases and Gene Function. J. Mol. Evol..

[26] Liu T., Xu Y., Wang X., Gu J., Yan G., Chen X.-G. (2018). Antiviral systems in vector mosquitoes. Dev. Comp. Immunol..

[27] Vabret N., Bhardwaj N., Greenbaum B.D. (2017). Sequence-Specific Sensing of Nucleic Acids. Trends Immunol..

[28] Netea M.G., Schlitzer A., Placek K., Joosten L.A.B., Schultze J.L. (2019). Innate and Adaptive Immune Memory: An Evolutionary Continuum in the Host’s Response to Pathogens. Cell Host Microbe.

[29] Merkling S.H., van Rij R.P. (2013). Beyond RNAi: Antiviral defense strategies in Drosophila and mosquito. J. Insect. Physiol..

[30] Fensterl V., Chattopadhyay S., Sen G.C. (2015). No Love Lost Between Viruses and Interferons. Annu. Rev. Virol..

[31] Halbach R., Junglen S., van Rij R.P. (2017). Mosquito-specific and mosquito-borne viruses: Evolution, infection, and host defense. Curr. Opin. Insect. Sci..

[32] Brackney D.E., Beane J.E., Ebel G.D. (2009). RNAi targeting of West Nile virus in mosquito midguts promotes virus diversification. PLoS Pathog..

[33] Grubaugh N.D., Smith D.R., Brackney D.E., Bosco-Lauth A.M., Fauver J.R., Campbell C.L., Felix T.A., Romo H., Duggal N.K., Dietrich E.A. (2015). Experimental evolution of an RNA virus in wild birds: Evidence for host-dependent impacts on population structure and competitive fitness. PLoS Pathog..

[34] Grubaugh N.D., Weger-Lucarelli J., Murrieta R.A., Fauver J.R., Garcia Luna S.M., Prasad A.N., Black W.C., Ebel G.D. (2016). Genetic Drift during Systemic Arbovirus Infection of Mosquito Vectors Leads to Decreased Relative Fitness during Host Switching. Cell Host Microbe.

[35] Grubaugh N.D., Ebel G.D. (2016). Dynamics of West Nile virus evolution in mosquito vectors. Curr. Opin. Virol..

[36] Brackney D.E., Schirtzinger E.E., Harrison T.D., Ebel G.D., Hanley K.A. (2015). Modulation of flavivirus population diversity by RNA interference. J. Virol..

[37] Nelson C.W., Sibley S.D., Kolokotronis S.-O., Hamer G.L., Newman C.M., Anderson T.K., Walker E.D., Kitron U.D., Brawn J.D., Ruiz M.O. (2018). Selective constraint and adaptive potential of West Nile virus within and among naturally infected avian hosts and mosquito vectors. Virus Evolut..

[38] Villordo S.M., Filomatori C.V., Sánchez-Vargas I., Blair C.D., Gamarnik A.V. (2015). Dengue Virus RNA Structure Specialization Facilitates Host Adaptation. PLoS Pathog..

[39] Li F., Zheng Q., Ryvkin P., Dragomir I., Desai Y., Aiyer S., Valladares O., Yang J., Bambina S., Sabin L.R. (2012). Global Analysis of RNA Secondary Structure in Two Metazoans. Cell Rep..

[40] Coffey L.L., Vignuzzi M. (2011). Host alternation of chikungunya virus increases fitness while restricting population diversity and adaptability to novel selective pressures. J. Virol..

[41] Vasilakis N., Deardorff E.R., Kenney J.L., Rossi S.L., Hanley K.A., Weaver S.C. (2009). Mosquitoes put the brake on arbovirus evolution: Experimental evolution reveals slower mutation accumulation in mosquito than vertebrate cells. PLoS Pathog..

[42] Ciota A.T., Lovelace A.O., Jones S.A., Payne A., Kramer L.D. (2007). Adaptation of two flaviviruses results in differences in genetic heterogeneity and virus adaptability. J. Gen. Virol..

[43] Jerzak G.V.S., Brown I., Shi P.-Y., Kramer L.D., Ebel G.D. (2008). Genetic diversity and purifying selection in West Nile virus populations are maintained during host switching. Virology.

[44] Greene I.P., Wang E., Deardorff E.R., Milleron R., Domingo E., Weaver S.C. (2005). Effect of alternating passage on adaptation of sindbis virus to vertebrate and invertebrate cells. J. Virol..

[45] Moutailler S., Roche B., Thiberge J.-M., Caro V., Rougeon F., Failloux A.-B. (2011). Host alternation is necessary to maintain the genome stability of rift valley fever virus. PLoS Negl. Trop. Dis..

[46] Novella I.S., Clarke D.K., Quer J., Duarte E.A., Lee C.H., Weaver S.C., Elena S.F., Moya A., Domingo E., Holland J.J. (1995). Extreme fitness differences in mammalian and insect hosts after continuous replication of vesicular stomatitis virus in sandfly cells. J. Virol..

[47] Novella I.S., Hershey C.L., Escarmis C., Domingo E., Holland J.J. (1999). Lack of evolutionary stasis during alternating replication of an arbovirus in insect and mammalian cells. J. Mol. Biol..

[48] Weaver S.C., Brault A.C., Kang W., Holland J.J. (1999). Genetic and fitness changes accompanying adaptation of an arbovirus to vertebrate and invertebrate cells. J. Virol..

[49] Ciota A.T., Kramer L.D. (2010). Insights into arbovirus evolution and adaptation from experimental studies. Viruses.

[50] Novella I.S., Presloid J.B., Smith S.D., Wilke C.O. (2011). Specific and Nonspecific Host Adaptation during Arboviral Experimental Evolution. J. Mol. Microbiol. Biotechnol..

[51] Jacobson G.N., Clark P.L. (2016). Quality over quantity: Optimizing co-translational protein folding with non-“optimal” synonymous codons. Curr. Opin. Struct. Biol..

[52] Křížek M., Křížek P. (2012). Why has nature invented three stop codons of DNA and only one start codon?. J. Theor. Biol..

[53] Burow D.A., Martin S., Quail J.F., Alhusaini N., Coller J., Cleary M.D. (2018). Attenuated Codon Optimality Contributes to Neural-Specific mRNA Decay in Drosophila. Cell Rep..

[54] Goldsmith M., i5K Consortium (2013). i5K Consortium The i5K Initiative: Advancing arthropod genomics for knowledge, human health, agriculture, and the environment. J. Hered..

[55] Rodriguez O., Singh B.K., Severson D.W., Behura S.K. (2012). Translational selection of genes coding for perfectly conserved proteins among three mosquito vectors. Infect. Genet. Evol..

[56] Whittle C.A., Extavour C.G. (2015). Codon and Amino Acid Usage Are Shaped by Selection Across Divergent Model Organisms of the Pancrustacea. Genes Genomes Genet..

[57] Abascal F., Posada D., Knight R.D., Zardoya R. (2006). Parallel Evolution of the Genetic Code in Arthropod Mitochondrial Genomes. PLoS Biol..

[58] Novoa E.M., Pavon-Eternod M., Pan T., Ribas de Pouplana L. (2012). A role for tRNA modifications in genome structure and codon usage. Cell.

[59] Tuorto F., Lyko F. (2016). Genome recoding by tRNA modifications. Open Biol..

[60] Behura S.K., Severson D.W. (2010). Coadaptation of isoacceptor tRNA genes and codon usage bias for translation efficiency in Aedes aegypti and Anopheles gambiae. Insect Mol. Biol..

[61] Parisien M., Wang X., Pan T. (2013). Diversity of human tRNA genes from the 1000-genomes project. RNA Biol..

[62] Di Giallonardo F., Schlub T.E., Shi M., Holmes E.C. (2017). Dinucleotide Composition in Animal RNA Viruses Is Shaped More by Virus Family than by Host Species. J. Virol..

[63] Behura S.K., Severson D.W. (2013). Codon usage bias: Causative factors, quantification methods and genome-wide patterns: With emphasis on insect genomes. Biol. Rev. Camb. Philos. Soc..

[64] Sharp P.M., Tuohy T.M., Mosurski K.R. (1986). Codon usage in yeast: Cluster analysis clearly differentiates highly and lowly expressed genes. Nucleic Acids Res..

[65] Peden J.F. (1999). Analysis of Codon Usage. Ph.D. Thesis.

[66] Pinheiro M., Afreixo V., Moura G., Freitas A., Santos M.A.S., Oliveira J.L. (2006). Statistical, computational and visualization methodologies to unveil gene primary structure features. Methods Inf. Med..

[67] Wright F. (1990). The “effective number of codons” used in a gene. Gene.

[68] Zaborske J.M., Bauer DuMont V.L., Wallace E.W.J., Pan T., Aquadro C.F., Drummond D.A. (2014). A Nutrient-Driven tRNA Modification Alters Translational Fidelity and Genome-wide Protein Coding across an Animal Genus. PLoS Biol..

[69] Schimmel P. (2018). The emerging complexity of the tRNA world: Mammalian tRNAs beyond protein synthesis. Nat. Rev. Mol. Cell Biol..

[70] Ran W., Higgs P.G. (2010). The Influence of Anticodon–Codon Interactions and Modified Bases on Codon Usage Bias in Bacteria. Mol. Biol. Evol..

[71] Smith B.L., Chen G., Wilke C.O., Krug R.M. (2018). Avian Influenza Virus PB1 Gene in H3N2 Viruses Evolved in Humans To Reduce Interferon Inhibition by Skewing Codon Usage toward Interferon-Altered tRNA Pools. MBio.

[72] Drolet B.S., van Rijn P., Howerth E.W., Beer M., Mertens P.P. (2015). A Review of Knowledge Gaps and Tools for Orbivirus Research. Vector Borne Zoonotic Dis..

[73] Simón D., Fajardo A., Sóñora M., Delfraro A., Musto H. (2017). Host influence in the genomic composition of flaviviruses: A multivariate approach. Biochem. Biophys. Res. Commun..

[74] Blitvich B.J., Firth A.E. (2017). A Review of Flaviviruses that Have No Known Arthropod Vector. Viruses.

[75] Jenkins G.M., Pagel M., Gould E.A., Paolo MD A., Holmes E.C. (2001). Evolution of Base Composition and Codon Usage Bias in the Genus Flavivirus. J. Mol. Evol..

[76] Singh N.K., Tyagi A. (2017). A detailed analysis of codon usage patterns and influencing factors in Zika virus. Arch. Virol..

[77] Moratorio G., Iriarte A., Moreno P., Musto H., Cristina J. (2013). A detailed comparative analysis on the overall codon usage patterns in West Nile virus. Infect. Genet. Evol..

[78] (2017). Stephanie Chan; Jing-hsiung Ou Hepatitis C Virus-Induced Autophagy and Host Innate Immune Response. Viruses.

[79] Sharma G., Raheja H., Das S. (2018). Hepatitis C virus: Enslavement of host factors. IUBMB Life.

[80] Chiari Y., Dion K., Colborn J., Parmakelis A., Powell J.R. (2010). On the Possible Role of tRNA Base Modifications in the Evolution of Codon Usage: Queuosine and Drosophila. J. Mol. Evol..

[81] Butt A.M., Nasrullah I., Qamar R., Tong Y. (2016). Evolution of codon usage in Zika virus genomes is host and vector specific. Emerg. Microbes Infect..

[82] Luo X., Liu Q., Xiong Y., Ye C., Jin D., Xu J. (2015). Genome-wide analysis of synonymous codon usage in Huaiyangshan virus and other bunyaviruses. J. Basic Microbiol..

[83] Gumpper R.H., Li W., Luo M. (2018). Constraints of viral RNA synthesis on codon usage of negative strand RNA virus. J. Virol..

[84] Jenkins G.M., Holmes E.C. (2003). The extent of codon usage bias in human RNA viruses and its evolutionary origin. Virus Res..

[85] Xu X., Fei D., Han H., Liu H., Zhang J., Zhou Y., Xu C., Wang H., Cao H., Zhang H. (2017). Comparative characterization analysis of synonymous codon usage bias in classical swine fever virus. Microb. Pathog..

[86] Wang M., Zhang J., Zhou J.-H., Chen H.-T., Ma L.-N., Ding Y.-Z., Liu W.-Q., Liu Y.-S. (2011). Analysis of codon usage in bovine viral diarrhea virus. Arch. Virol..

[87] Schweizer M., Peterhans E. (2014). Pestiviruses. Annu. Rev. Anim. Biosci..

[88] Keller B.C., Johnson C.L., Erickson A.K., Gale M. (2007). Innate immune evasion by hepatitis C virus and West Nile virus. Cytokine Growth Factor Rev..

[89] Chen S.L., Lee W., Hottes A.K., Shapiro L., McAdams H.H. (2004). Codon usage between genomes is constrained by genome-wide mutational processes. Proc. Natl. Acad. Sci. USA.

[90] Knight R.D., Freeland S.J., Landweber L.F. (2001). A simple model based on mutation and selection explains trends in codon and amino-acid usage and GC composition within and across genomes. Genome Biol..

[91] Belalov I.S., Lukashev A.N. (2013). Causes and Implications of Codon Usage Bias in RNA Viruses. PLoS ONE.

[92] Burns C.C., Shaw J., Campagnoli R., Jorba J., Vincent A., Quay J., Kew O. (2006). Modulation of poliovirus replicative fitness in HeLa cells by deoptimization of synonymous codon usage in the capsid region. J. Virol..

[93] Burns C.C., Campagnoli R., Shaw J., Vincent A., Jorba J., Kew O. (2009). Genetic inactivation of poliovirus infectivity by increasing the frequencies of CpG and UpA dinucleotides within and across synonymous capsid region codons. J. Virol..

[94] Song Y., Gorbatsevych O., Liu Y., Mugavero J., Shen S.H., Ward C.B., Asare E., Jiang P., Paul A.V., Mueller S. (2017). Limits of variation, specific infectivity, and genome packaging of massively recoded poliovirus genomes. Proc. Natl. Acad. Sci. USA.

[95] Velazquez-Salinas L., Risatti G.R., Holinka L.G., O’Donnell V., Carlson J., Alfano M., Rodriguez L.L., Carrillo C., Gladue D.P., Borca M.V. (2016). Recoding structural glycoprotein E2 in classical swine fever virus (CSFV) produces complete virus attenuation in swine and protects infected animals against disease. Virology.

[96] Takata M.A., Soll S.J., Emery A., Blanco-Melo D., Swanstrom R., Bieniasz P.D. (2018). Global synonymous mutagenesis identifies cis-acting RNA elements that regulate HIV-1 splicing and replication. PLoS Pathog..

[97] Martínez M.A., Jordan-Paiz A., Franco S., Nevot M. (2016). Synonymous Virus Genome Recoding as a Tool to Impact Viral Fitness. Trends Microbiol..

[98] Stauft C.B., Shen S.H., Song Y., Gorbatsevych O., Asare E., Futcher B., Mueller S., Payne A., Brecher M., Kramer L. (2018). Extensive recoding of dengue virus type 2 specifically reduces replication in primate cells without gain-of-function in Aedes aegypti mosquitoes. PLoS ONE.

[99] Li P., Ke X., Wang T., Tan Z., Luo D., Miao Y., Sun J., Zhang Y., Liu Y., Hu Q. (2018). Zika Virus Attenuation by Codon Pair Deoptimization Induces Sterilizing Immunity in Mouse Models. J. Virol..

[100] Wang B., Yang C., Tekes G., Mueller S., Paul A., Whelan S.P.J., Wimmer E. (2015). Recoding of the vesicular stomatitis virus L gene by computer-aided design provides a live, attenuated vaccine candidate. MBio.

[101] Nougairede A., De Fabritus L., Aubry F., Gould E.A., Holmes E.C., de Lamballerie X. (2013). Random Codon Re-encoding Induces Stable Reduction of Replicative Fitness of Chikungunya Virus in Primate and Mosquito Cells. PLoS Pathog..

[102] Kunec D., Osterrieder N. (2016). Codon Pair Bias Is a Direct Consequence of Dinucleotide Bias. Cell Rep..

[103] Simmonds P., Tulloch F., Evans D.J., Ryan M.D. (2015). Attenuation of dengue (and other RNA viruses) with codon pair recoding can be explained by increased CpG/UpA dinucleotide frequencies. Proc. Natl. Acad. Sci. USA.

[104] Atkinson N.J., Witteveldt J., Evans D.J., Simmonds P. (2014). The influence of CpG and UpA dinucleotide frequencies on RNA virus replication and characterization of the innate cellular pathways underlying virus attenuation and enhanced replication. Nucleic Acids Res..

[105] de Visser J.A.G.M., Hermisson J., Wagner G.P., Meyers L.A., Chaichian H.B., Blanchard J.L., Chao L., Cheverud J.M., Elena S.F., Fontana W. (2003). Perspective: Evolution and Detection of Genetic Robustness. Evolution.

[106] Fares M.A. (2015). The origins of mutational robustness. Trends Genet..

[107] Lachowiec J., Queitsch C., Kliebenstein D.J. (2016). Molecular mechanisms governing differential robustness of development and environmental responses in plants. Ann. Bot..

[108] Sanjuán R. (2010). Mutational fitness effects in RNA and single-stranded DNA viruses: Common patterns revealed by site-directed mutagenesis studies. Philos. Trans. R. Soc. B Biol. Sci..

[109] Belshaw R., Gardner A., Rambaut A., Pybus O.G. (2008). Pacing a small cage: Mutation and RNA viruses. Trends Ecol. Evol..

[110] Ochsenreiter R., Hofacker I.L., Wolfinger M.T. (2019). Functional RNA Structures in the 3′UTR of Tick-Borne, Insect-Specific and No-Known-Vector Flaviviruses. Viruses.

[111] Prostova M.A., Gmyl A.P., Bakhmutov D.V., Shishova A.A., Khitrina E.V., Kolesnikova M.S., Serebryakova M.V., Isaeva O.V., Agol V.I. (2015). Mutational robustness and resilience of a replicative cis-element of RNA virus: Promiscuity, limitations, relevance. RNA Biol..

[112] Rodrigo G., Daròs J.-A., Elena S.F. (2017). Virus-host interactome: Putting the accent on how it changes. J. Proteomics.

[113] Lauring A.S., Acevedo A., Cooper S.B., Andino R. (2012). Codon usage determines the mutational robustness, evolutionary capacity, and virulence of an RNA virus. Cell Host Microbe.

[114] Burch C.L., Chao L. (2000). Evolvability of an RNA virus is determined by its mutational neighbourhood. Nature.

[115] Dolan P.T., Whitfield Z.J., Andino R. (2018). Mapping the Evolutionary Potential of RNA Viruses. Cell Host Microbe.

[116] Braun T., Bordería A.V., Barbezange C., Vignuzzi M., Louzoun Y. (2018). Long term context dependent genetic adaptation of the viral genetic cloud. Bioinformatics.

[117] Lauring A.S., Frydman J., Andino R. (2013). The role of mutational robustness in RNA virus evolution. Nat. Rev. Microbiol..

[118] Elena S.F. (2012). RNA virus genetic robustness: Possible causes and some consequences. Curr. Opin. Virol..

[119] Wilke C.O., Wang J.L., Ofria C., Lenski R.E., Adami C. (2001). Evolution of digital organisms at high mutation rates leads to survival of the flattest. Nature.

[120] Rihn S.J., Hughes J., Wilson S.J., Bieniasz P.D. (2015). Uneven genetic robustness of HIV-1 integrase. J. Virol..

[121] Visher E., Whitefield S.E., McCrone J.T., Fitzsimmons W., Lauring A.S. (2016). The Mutational Robustness of Influenza A Virus. PLoS Pathog..

[122] Stern A., Bianco S., Te Yeh M., Wright C., Butcher K., Tang C., Nielsen R., Andino R. (2014). Costs and Benefits of Mutational Robustness in RNA Viruses. Cell Rep..

[123] Cuevas J.M., Moya A., Sanjuan R. (2009). A genetic background with low mutational robustness is associated with increased adaptability to a novel host in an RNA virus. J. Evol. Biol..

[124] Warmbrod K.L., Patterson E.I., Kautz T.F., Stanton A., Rockx-Brouwer D., Kalveram B.K., Khanipov K., Thangamani S., Fofanov Y., Forrester N.L. (2019). Viral RNA-dependent RNA polymerase mutants display an altered mutation spectrum resulting in attenuation in both mosquito and vertebrate hosts. PLoS Pathog..

