# Supplementary material for: Effects of Arbovirus Multi-Host Life Cycles on Dinucleotide and Codon Usage Patterns
Source: Viruses. 2019 Jul 12;11(7):643. doi: 10.3390/v11070643 (PMC6669465; doi:10.3390/v11070643)
Supplement: Supplementary file 1 [file viruses-11-00643-s001.pdf]

Second Base of Codon

First Base of Codon	U		C		A		G		Third Base of Codon
U	UUU	Phenylalanine (F)	UCU	Serine (S)	***UAU***	Tyrosine (Y)	***UGU***	Cysteine (C)	U
	UUC		UCC		***UAC***		***UGC***		C
	UUA	Leucine (L)	***UCA***		UAA	STOP	UGA	STOP	A
	UUG		***UCG***		UAG		***UGG***	Tryptophan (W)	G
C	CUU	Leucine (L)	CCU	Proline (P)	CAU	Histidine (H)	CGU	Arginine (R)	U
	CUC		CCC		CAC		CGC		C
	CUA		CCA		***CAA***	Glutamine (Q)	***CGA***		A
	CUG		CCG		***CAG***		CGG		G
A	AUU	Isoleucine (I)	ACU	Threonine (T)	AAU	Asparagine (N)	AGU	Serine (S)	U
	AUC		ACC		AAC		AGC		C
	AUA		ACA		***AAA***	Lysine (K)	***AGA***	Arginine (R)	A
	AUG	Methionine (M)	ACG		***AAG***		AGG		G
G	GUU	Valine (V)	GCU	Alanine (A)	GAU	Aspartic Acid (D)	GGU	Glycine (G)	U
	GUC		GCC		GAC		GGC		C
	GUA		GCA		***GAA***	Glutamic Acid (E)	***GGA***		A
	GUG		GCG		***GAG***		GGG		G

Supplementary Figure 1. Codon usage table with one-to-stop (OTS) codons. Stop codons are highlighted in red and OTS codons are indicated with bold italicized letters.
